# Teaming beyond the clinical environment – building collective commitment across graduate medical education

**DOI:** 10.1186/s12909-023-04713-3

**Published:** 2023-10-18

**Authors:** Brady S. Laughlin, Elaine M. Griffeth, Aaron F. Bush, Cheryll A. Albold, Christopher J. Boes, Annie T. Sadosty

**Affiliations:** 1 Mayo Clinic School of Graduate Medical Education, Mayo Clinic College of Medicine and Science, 5881 E Mayo Blvd, Phoenix, AZ USA; 2 Mayo Clinic School of Graduate Medical Education, Mayo Clinic College of Medicine and Science, Rochester, MN USA; 3 Mayo Clinic School of Graduate Medical Education, Mayo Clinic College of Medicine and Science, Jacksonville, FL USA

## Abstract

This commentary provides evidence and expert opinion on effective relationships and communication strategies for trainee and graduate medical education leaders. The authors also argue that consistent communication and alignment of goals between trainee leadership and graduate medical education leadership are essential components of a successful collaboration that promotes trainee well-being.

As health-care worker well-being has become an important area of research, graduate medical education (GME) has increasingly valued the clinical learning environment and trainee experience as important factors in achieving the goal result of safe and competent board-certified physicians [1–5]. Furthermore, the COVID-19 pandemic impacted the clinical learning environment in unprecedented ways, highlighting pre-existing issues in GME such as burnout, social isolation, and fatigue [6–9]. GME leaders must work in partnership with trainee, also known as house staff, leaders to promote wellness in the workplace. Unions have been formed at some institutions to be the exclusive representative negotiating on behalf of residents and fellows, while other institutions have maximized relationships with existing local trainee associations [10–12]. In this article, we discuss a strategic framework for collaboration and collective commitment across GME within the context of teaming, as well as illustrate a case example from our institutional experience between GME leadership and a voluntary resident and fellow association (Fig. 1).

**Fig. 1:**
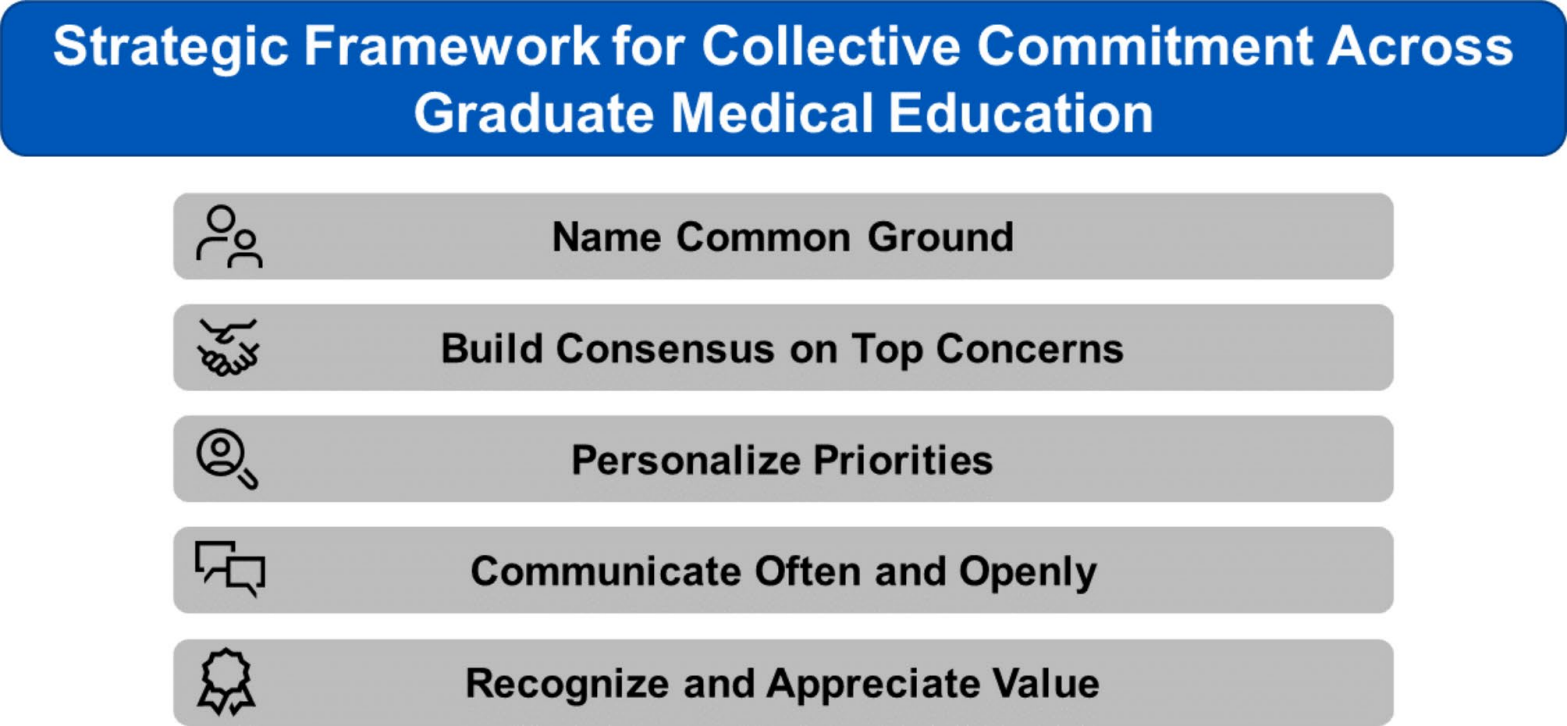
Strategic Framework for Collective Commitment Across Graduate Medical Education

The Mayo Clinic School of Graduate Medical Education, established in 1915, has more than 1800 trainees in 350 residency and fellowship programs across three distinct geographical regions – the Midwest, Florida, and Arizona. GME leaders are dedicated to the success of the residency and fellowship programs and maintaining compliance with the Accreditation Council for Graduate Medical Education (ACGME) institutional and program requirements. Advancing the quality of resident and fellow education through accreditation, as is the mission of the ACGME, relies on not only GME leaders but also trainee insights and perspectives [13]. Therefore, it is critical that trainees have a pathway to effectively communicate needs and goals without fear of negative consequences. The Mayo Fellows’ Association (MFA), established in 1930, is a voluntary membership association open to all residents and fellows at Mayo Clinic that has chapters in Florida, Arizona, and the Midwest. The elected presidents of these three chapters work closely with trainees at their sites to identify and understand needs, advocate on behalf of trainees, promote wellness, and enhance connection across programs. The collective trainee voice that leaders of voluntary house staff associations represent is an essential element of a healthy GME environment.

## Case example

Burnout and fatigue are important factors that play a significant role in the training of graduate medical learners [1]. The COVID-19 pandemic has caused significant disruption in the medical training of residents and fellows. To understand the needs of the trainees, surveys were distributed to trainees in Mayo Clinic GME programs in the three geographies, Arizona, Florida, and the Midwest in the summer of 2022 by Mayo Fellows’ Association leaders. Survey data indicated a strong desire for an increase in the number of vacation days to improve wellness needs of graduate medical trainees. The total number of vacation days per year at that time was 15. The data also demonstrated a strong desire for stipend increases. In addition to the survey, site specific townhalls were held with Mayo Clinic GME leadership during summer of 2022 to allow trainees to directly communicate their concerns. During these townhalls, trainees brought forward personal stories regarding cost of living challenges with their current stipend level. . These personal accounts providedMFA leaders and GME leadership an opportunity to collectively understand the personal impact of the current stipend and vacation policy and the needs of the trainees.

Following the townhalls, MFA leaders and Mayo Clinic GME leaders engaged in discussions to establish top priorities and addressed each over the ensuing academic year through a five-step framework (Fig. 1). This framework was a new approach to GME improvement efforts at our institution. Previously, GME leaders identified areas for improvement by working with training program directors and by soliciting input from trainee representatives during regularly scheduled graduate education committee meetings. Mayo Fellows’ Association leaders often serve as those representatives, and their input was frequently solicited as to suggestions for specific topics for improvement. In addition, the MFA leaders routinely met with the site specific graduate education committee as well as enterprise GME leaders to bring up concerns.

Prior to the COVID-19 pandemic, there was consideration of increasing vacation days, which required extensive research including benchmarking and determining the impact of absence from training on qualification for board certification. The Graduate Medical Education Committee made the difficult decision to not increase vacation days when the impact of the COVID-19 pandemic became clear. As time has moved forward, GME leaders and the MFA have collaborated with focuses on improving trainee well-being and expanding the reputation of the Mayo Clinic training programs.

To better target top concerns, our institution’s GME culture shifted to a collaborative approach using the five-step framework. The trainee and GME leaders ***named common ground*** by identifying goals of increasing the desirability of Mayo Clinic’s training programs and supporting trainee well-being. After identifying these goals, a ***consensus was built*** on increasing stipend and vacation to improve trainee wellness and satisfaction. A work group was formed, and frequent meetings occurred between the trainee and GME leaders to ensure there was progress on these priorities. A benchmarking analysis of stipends at both geographic and national peer programs was performed. This led to the introduction of a 6% market-based stipend adjustment. Each year, stipend increases are evaluated and alignment is considered with institutional percentages for allied health. GME leaders advocated for equity for trainees. The workgroup also benchmarked total vacation days among those same programs and evaluated the impact of the proposed vacation increase on board certification, current trip policy, and required case logs. Core GME leaders understood the personal impact of an increase in stipend and vacation days from participating in one-on-one meetings with MFA leaders, graduate education committee meetings, and town halls, and trainee and GME leaders shared stories of the personal impact of this change during presentations to the wider GME stakeholder community to ***personalize these priorities***.

The workgroup met frequently to review opinions and concerns of the wider GME stakeholder community. This ***often and open communication*** allowed for both trainee and GME leaders to work on not only writing the policy change but also form suggestions for implementation that addressed concerns regarding board eligibility among various specialties. Processes of the workgroup included review of the current vacation and trip policy and discussion and proposal of new policies. The workgroup reviewed national GME specialty board eligibility requirements. Trainee compensation and benefit time off plans were evaluated. The workgroup solicited further input from program directors and other key stakeholders to create a draft of policy. Initial polling of the policy at site specific graduate education meetings was performed. Positive and constructive feedback was gathered, and used to generate a final version of the policy. Following finalization of an official policy, this policy was proposed for approval to the Mayo Clinic Graduate Medical Education Committee.

With introduction of a market-based stipend adjustment and successful approval of the new vacation policy, which increased vacation from 15 to 20 days for all trainees at Mayo Clinic training programs, trainees feel ***recognized and appreciated for the value*** they contribute to the institution. Additionally, this five-step process for GME improvement efforts strengthened relationships between trainee leaders and GME leaders and improved the collaborative landscape and culture of GME.

### Name common ground

The first step in our proposed framework is to name common ground. When learners, employees, and leadership all take ownership of an institution’s mission, that mission can be a powerful unifying force [14]. The primary value at Mayo Clinic, for example, is very clear: “The needs of the patient come first.” This shared value naturally forms a team across all roles as individuals work together to meet the needs of patients. The concept of teaming, which has been included as a pathway to excellence for the clinical learning environment, highlights the importance of all individuals coming together and using their different professional strengths to achieve a common vision and goals [15]. This concept can be applied beyond patient care as GME and trainee leaders collaborate on and share accountability for their institution’s clinical learning environment and trainee experience [15]. These leaders share a commitment to promoting trainee wellness and achieving outstanding results from their programs. While hierarchy may be inherent in GME, naming common ground or a mission statement that unifies rather than stratifies the team not only improves relationships between GME and trainee leaders but also builds a collective commitment to balancing patient care, education, and working conditions.

### Build consensus on top concerns

Once shared values have been emphasized and common ground has been named, GME and trainee leaders can then work together to identify concerns and build consensus on a set of priorities. Institutional GME leaders work to identify best practices and strategic initiatives that will advance the quality of its educational programs. Similarly, leaders of voluntary house staff associations work to identify and understand needs and advocate on behalf of trainees. In our experience, anonymous surveys have been successful in capturing the trainee voice as they provide an opportunity to perform a comprehensive needs assessment and evaluation of current resources. However, as the trainee experience differs across specialties and geographies, it is important to have survey components that allow for appropriate subgroup analysis to more accurately characterize trainee needs while maintaining anonymity. Trainee leaders can then use the data from surveys as well as the context of their own experiences to advocate effectively for these concerns. Once GME and trainee leaders have identified opportunities for improvement in the clinical learning environment and trainee experience, they can then build consensus on a set of priorities. Open discussion provides an opportunity to learn from team members’ experience, understand the impact a potential change could make, and focus on priority areas where goals of trainees and GME align [16].

### Personalize priorities

Leaders must communicate effectively in order to build motivation for change [14]. The next step in the framework is to personalize the priorities set when building consensus. Storytelling is an effective approach, framing issues within familiar context for the listener and engaging their sense of empathy [14, 17, 18]. Leaders must be able to highlight personal stories behind the needs and concerns the priorities address so that stakeholders also desire the changes suggested. These stories can be communicated anonymously by leaders or by individuals themselves in open forums like town halls. In addition, leaders need to build belief that the plans will be successful in promoting wellness by addressing the existing gap in the clinical learning environment and/or trainee experience. Studies demonstrating effective workplace interventions to promote wellness have been limited, so finding ways to communicate the personal impact of proposed changes may be more successful [2, 3, 19–21].

### Communicate often and openly

Once common ground has been named and priorities have been set, it is important for GME and trainee leaders to communicate often and openly. Regularly scheduled meetings with thoughtful and active discussion are important for understanding these priorities within the larger institutional culture, making decisions, and delegating responsibilities so that progress can be made on the team’s goals and priorities throughout the academic year [22]. Team members should expect to come to meetings as interested and invested listeners with open minds [15]. Email and touchpoint meetings on an as-needed basis can help maintain momentum throughout the year, address differences across specialties as regards board requirements, and overcome any unforeseen challenges. Another facet of this communication is trainee leader participation on institutional and GME committees alongside their GME leader counterparts. The more the trainee leaders can be aware of institutional changes and challenges, the better informed they can be as team members in GME.

Frequent and transparent communication with the trainee community as a whole is also important. The priorities and plans for the academic year should be communicated once they’ve been established as well as progress made at the end of the year. This communication should incorporate the institution’s mission statement (naming common ground) and should come from the team of GME and trainee leaders. Trainees appreciate direct lines of communication with GME leaders, and email, townhalls, and question and answer sessions have been shown to be successful [7].

### Recognize and appreciate value

The last component of this framework is to recognize and appreciate the value of trainees as well as those who work on their behalf. Trainees are essential members of the clinical team and education mission of medical schools. Recently, the Gold Humanism Honor Society initiated “Thank a Resident Day” in 2018 to recognize the contributions of trainees [23]. Institutions and GME should collaborate with voluntary house staff associations to use that opportunity to recognize the value added by trainees and show appreciation. Similarly, faculty invested in GME leadership positions should be recognized for their work on behalf of trainees by their departments and the institution. When highly motivated and thoughtful people are in those positions, trainees’ benefit. Additionally, including trainees on hospital and GME committees communicates an institutional commitment to valuing the trainee perspective and its educational programs.

## Discussion

House staff associations play an important role in the landscape of graduate medical education. Medical trainees are often represented by elected residents/fellows who serve as leaders of a council or union. The role of a house staff association is to advocate for the needs of an institution’s trainees. As the representatives of all residency and fellowship program members at an institution, the house staff association acts as a forum to promote advocacy and social connection. At Mayo Clinic, the Mayo Fellows’ Association serves as the structure to channel feedback from trainees and translate that into top priorities for discussion with GME leaders.

In the setting of graduate medical education, collaboration between trainee leaders and GME leaders may seem like an easy task, but it is not quite that simple. A hospital/education center’s GME culture provides the pathway to be able to engage in discussions between these two groups [22]. However, a hospital’s GME culture is multi-faceted and often complex, deriving from hierarchies, teams, interconnected professions, and subcultures [22]. An impactful difference of the framework established above compared to other communication frameworks is that its collaborative approach relies on listening. By adopting strategies of co-leadership, GME can create effective relationships with trainees. Strategies such as consensus building and enhanced communication are essential to establishing priorities and initiating effective improvement efforts – efforts which ultimately positively impact the GME training experience.

In conclusion, graduate medical education is most successful when GME and trainee leaders see themselves as a team and share a collective commitment to improving the clinical learning environment and trainee experience. Effective relationships between these groups allow an institution to meet trainee needs consistently and effectively under normal conditions as well as in times of crisis. The framework suggested in this article (name common ground, build consensus on top concerns, personalize priorities, communicate often and openly, and recognize and appreciate value) can serve as a guide for building a team across all stakeholders in graduate medical education.

## Data Availability

Not applicable.
